# Macular pigment in retinal health and disease

**DOI:** 10.1186/s40942-016-0044-9

**Published:** 2016-08-15

**Authors:** Verônica Castro Lima, Richard B. Rosen, Michel Farah

**Affiliations:** 1 Retina Service, Hospital Humberto Castro Lima (IBOPC), Salvador, Bahia Brazil; 2 Retina Service, Department of Ophthalmology, Federal University of São Paulo, São Paulo, Brazil; 3 Icahn School of Medicine at Mount Sinai, New York, NY USA; 4 Retina Service, Department of Ophthalmology, The New York Eye and Ear Infirmary of Mount Sinai, 310 East 14th Street, New York, NY 10003 USA

## Abstract

Lutein and zeaxanthin, two carotenoid pigments of the xanthophyll subclass, are present in high concentrations in the retina, especially in the macula. They work as a filter protecting the macula from blue light and also as a resident antioxidant and free radical scavenger to reduce oxidative stress-induced damage. Many observational and interventional studies have suggested that lutein and zeaxanthin may reduce the risk of various eye diseases, especially late forms of AMD. In vitro and in vivo studies indicate that they could protect various ocular cells against oxidative damage. Recent research has shown that in addition to traditional mechanisms, lutein and zeaxanthin can influence the viability and function of cells through various signal pathways or transcription factors: for instance, they can affect immune responses and inflammation, and have anti-angiogenic and anti-tumor properties. This review covers the basic aspects and results of recent studies regarding the effects of lutein, zeaxanthin and other carotenoids, such as meso-zeaxanthin, on the eye in different clinical and experimental models and the management of various ocular diseases using these molecules.

## Background

**Fig. 1:**
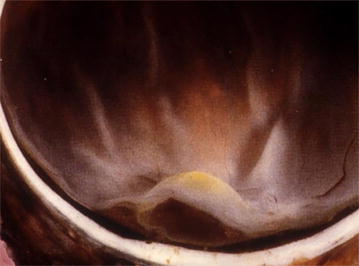
Autopsy specimen of the macula lutea or “yellow spot”

The central portion of the retina or macula is responsible for optimal spatial vision [1]. Macular pigment (MP) is a generic term used to describe the yellow pigment composed principally of the three isomeric carotenoids meso-zeaxanthin (MZ), lutein (L), and zeaxanthin (Z), which accumulate in the macula [2, 3] (Fig. 1). The highest levels of MP in the human body are measured in Henle’s fibers at the fovea and in the inner nuclear layer in the parafoveal area [4–6]. MP acts as an optical filter for blue light and provides antioxidant protection to the human retina by inhibiting the peroxidation of long-chain polyunsaturated fatty acids [7–9]. Thus, the anatomical [2], biochemical [8], and optical [7] properties of MP have generated interest in its role in vision and macular health.

There is increasing evidence that MP is important for vision in normal subjects [10, 11], based upon MP’s ability to optimize visual performance and experience by attenuating chromatic aberration, veiling luminance, and blue haze [12]. Moreover, it has generated interest because of its possible protective role against age-related macular degeneration (AMD), the western world’s leading cause of irreversible blindness [13]. Since oxidative damage seems to be an important factor for the development and exacerbation of some retinal diseases [14, 15], the postulated protective role of MP in some disorders, especially AMD, has been extensively investigated over the past decades [16, 17].

## Anatomical, chemical and optical properties of MP related to their biofunctions in the eye

Macular pigment concentration peaks at the foveola. L is the dominant carotenoid in the peripheral macula, Z in the mid-peripheral macula, and MZ at the epicenter of the macula [2, 5, 6]. The term macula lutea (yellow spot) is actually attributable to the presence of these carotenoids in the central region of the retina [12]. The L:Z ratio in the fovea is approximately 1:2.4. Moving eccentrically to the periphery, Z levels decline while L levels increase. Therefore, L:Z in the peripheral macula reverses, exceeding 2:1 [18]. Bone et al. demonstrated that Z coexists with its isomer MZ in the fovea. They also proposed that L, MZ, and Z are actually found in equal quantities in the central macula (area 3 mm in diameter within the central macula) [19]. MZ, unlike L and Z, was previously thought to be undetectable in the human liver or serum. Thus, it was theorized that MZ was a specific metabolite of L found only in the retina. The L:Z of 3:1 in serum and 2:1 in the fovea support the theory of conversion of L to MZ in the macula [2]. However, more recently, MZ has been detected in serum, and supplementation trials have demonstrated a significant increase in macular pigment levels after oral administration of MZ, suggesting that MZ can be absorbed after oral intake and transported to the macula [20–22]. In addition, the augmentation of macular pigment was higher after oral administration of formulas containing MZ [22].

**Fig. 2:**
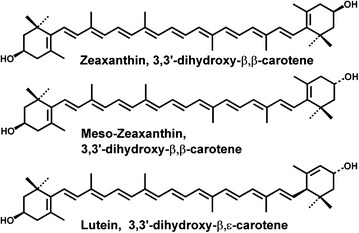
Biochemical structures of the main carotenoids of macular pigment

Carotenoids are often referred to as pigments or chromophores because of their mostly colored nature and consequential ability to absorb visible light. All carotenoids have a characteristic linear conjugated polyene chain and are classed into one of two subgroups. The hydrocarbon carotenoids are known as carotenes, such as β-carotene and lycopene, whereas carotenoids that are substituted with hydroxyl (–OH) functional groups are known as xanthophylls and include L, Z and MZ [23]. The hydroxyl functional groups permit L, Z and their structural isomers to cross the blood–ocular and blood–brain barriers. Other carotenoids (β-carotene and lycopene) contain only carbon and hydrogen atoms and do not cross the blood–brain or ocular barriers [24, 25]. β-carotene only crosses the blood brain barrier after it is cleaved to form retinaldehyde and other metabolites [26]. L and Z share the carbon skeleton and bonding framework of a- and b-carotene, respectively. The bonding frameworks of these two carotenoids may appear identical, at first glance. The chemical formulas of L and Z are chemically distinguished from one another in important ways (Fig. 2). Z exists in three stereoisomeric forms, the result of the two stereocenters at carbons 3 and 3′, the sites of the secondary hydroxyl groups. L can exist in eight stereoisomeric forms as a result of the presence of three stereocenters at the 3, 3′, and 6′ carbon atoms. In addition, the hydroxyl group at carbon 3′ of L is allylic [3]. Z and MZ are classified as diastereomers and differ only in the spatial orientation of the hydroxyl group on the C3′ chiral position. The structural differences of MZ, L, and Z have important implications for their respective antioxidant and light-filtering properties [23, 27].

The absorption spectra of MP peak at 460 nm [28, 29]. Although MZ, L, and Z exhibit very similar absorption spectra, it is possible to distinguish them on the basis of discrete variations in relative absorbance (nm) and intensity (AU) [3]. Because of their chemical structure, these carotenoids together are responsible for blue-light absorption and quenching of reactive oxygen species (ROS), thus attenuating oxidative injury at the macula [23, 30, 31]. Although Z and MZ are more efficient than L at eliminating ROS, a mixture of L, Z, and MZ in vitro, at a ratio of 1:1:1, has been shown to quench more singlet oxygen than any of these individual carotenoids at the same total concentration [32–34]. Macular carotenoids are very effective antioxidants, capable of quenching singlet oxygen and triplet state photosensitizers, inhibiting peroxidation of membrane phospholipids, scavenging ROS, and reducing lipofuscin formation [35, 36]. These essential functions of macular pigment decrease oxidative stress in the retina and enhance vision in both normal and abnormal retinas.

## Macular pigment and visual performance

**Fig. 3:**
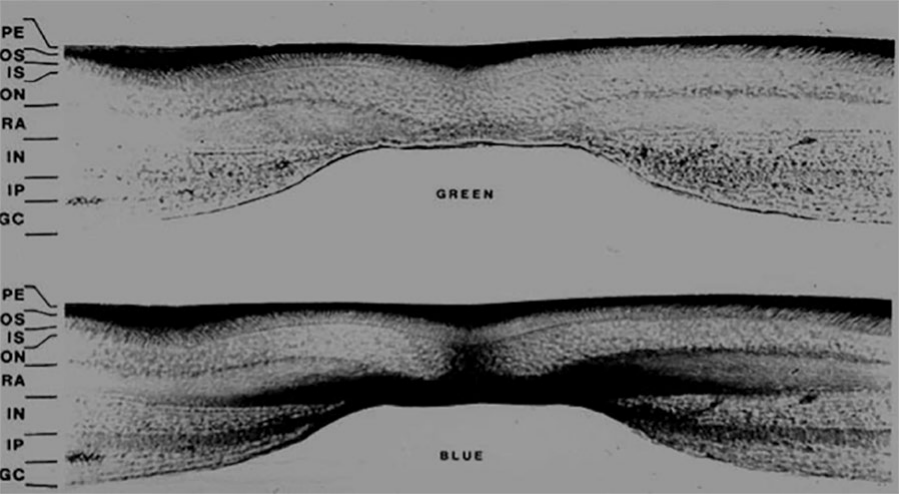
Differential absorption of blue light shows MP location in the Henle fiber layer and inner plexiform layer along the axons of the photoreceptors

Macular pigment enhances visual function in a variety of ways. The filtration of blue light (400–500 nm) reduces chromatic aberration, which can enhance visual acuity and contrast sensitivity (CS) (Fig. 3). L and Z also reduce discomfort associated with glare and improve photostress recovery time, macular function, and neural processing speed. Discomfort glare is a term used to describe photophobia and discomfort experienced when intense light enters the eye. Stringham et al. analyzed the photophobic response in normal subjects and found that those with higher MP levels tolerated xenon light better. They concluded that light containing short-wavelength energy appeared to be especially discomforting and that MP appeared to act as a spatially integrated filter, serving to attenuate photophobia to a great extent [37]. Similarly, Wenzel et al. showed a direct correlation between MP level and photophobia threshold [38].

Disability glare is a term used to describe decreased visual acuity resulting from scattered light, another phenomenon that results from bright light settings. Photostress recovery is described as the time necessary to recover vision following exposure to a bright light source. Physiologically, it represents the time necessary for photopigments bleached by a bright light source to regenerate. A study conducted by Stringham and Hammond [39] demonstrated that subjects with higher MP levels maintained better visual acuity than subjects with lower levels when exposed to both bright white light and short wavelength (blue) light. Additionally, photostress recovery time, after exposure to xenon-white light, was significantly shorter for subjects with higher MP levels. A more recent study showed that higher MP optical densities (MPODs) resulted in faster photostress recovery times, lower disability glare contrast thresholds, and lower visual discomfort [40]. Hammond et al. reported that daily oral supplementation with L (10 mg/day) and Z (2 mg/day) in 57 patients for 12 months resulted in a significant increase in serum levels of L and Z and in MPOD and significantly improved chromatic contrast and photostress recovery time [41]. These results were consistent with past studies showing that increasing MPOD leads to improved visual performance.

Nolan et al. performed a randomized, placebo-controlled clinical trial supplementing healthy and young subjects with lutein and zeaxanthin for 12 months [42]. The goal was to identify if different parameters of visual performance could be improved after supplementation in a population with relatively high macular pigment levels who are considered to be at peak visual performance. The authors did not show a significant change in visual performance in supplemented patients despite increasing serum L levels and central macular pigment levels. However, they did demonstrate significant differences in mesopic CS and light/dark adaptation in a comparative analysis between the lower and the upper MP tertile groups. These findings suggest that subjects with low MP levels and suboptimal visual performance may benefit more from supplementation.

Interestingly, several studies have reported, among normal subjects, findings suggesting that MP may play a key role in visual health through a complex interplay between the optical, neurological and physiological mechanisms underlying vision. These observations include a better critical flicker fusion frequency in the presence of higher MPOD [43], associations between high MPOD and crystalline lens transparency and cataract formation [44], the presence of L and Z at substantial concentrations in the primary visual cortex, [45] and higher pattern electroretinogram P50 amplitudes and better dark adapted cone sensitivities in association with higher MPOD [46]. Additionally, MPOD correlates with processing speed and cognitive performance in healthy elderly subjects as well as those with mild cognitive impairment [47–49]. Another study found moderate but statistically significant improvements in both MPOD and visual processing speed when supplementing young and healthy individuals considered to be at peak cognitive efficiency [50].

## Clinical measurement of MP

Several objective techniques have been used to measure MPOD indirectly and noninvasively in vivo. They are divided into either psychophysical (heterochromatic flicker photometry, HFP [51] and motion photometry [52]) or optical methods (autofluorescence spectrometry [53], reflectometry [54], imaging reflectometry [55] and Raman spectrometry [56]). They have been previously well described in the literature and each one has certain merits and limitations. In conjunction with many of the MPOD techniques, HFP is based on the spectral absorption properties and retinal location of MP. Essentially, HFP determines MPOD by presenting a light stimulus of two alternating wavelengths at the fovea and at a parafoveal area, one that is short and maximally absorbed by MP, and another longer and not absorbed by MP [57]. This method has evolved during the past decades and it has been used in different study and clinical populations. Its main disadvantage is the difficulty to perform in young patients, people with insufficient visual acuity or visual fields, or with low cognitive tasks.

The autofluorescence method is based on the principle of autofluorescence of lipofuscin, which is located in the retinal pigment epithelium cells. This is one of the more recently introduced methods and allows objective and reliable measurements of MP distribution in the retina and is particularly easy to implement in a clinical practice [58–61]. Quantitative measurement of light reflected from the fundus is known as fundus reflectometry and its main goal is compare light reflected at the macula and light reflected from a peripheral area of the retina. Currently, HFP and retinal reflectometry are the most widely used techniques for MPOD measurement in the research and clinical environments, respectively [57].

## Clinical effects of supplementation of lutein and zeaxanthin in patients with AMD

AMD is a chronic, progressive, degenerative eye disease affecting the central retina, responsible for highest visual acuity. It has become the major cause of legal blindness in the elderly, in both developed and developing countries [62–64]. It has been shown that oxidative stress related to low-grade inflammation and hypoxia in the outer retina are important in the pathogenesis of AMD [62]. This is consistent with the idea that low MPOD may be a risk factor for AMD, because the macular carotenoids show potent properties as antioxidants [9, 65].

Indeed, many large observational studies have shown an inverse relationship between dietary intake of L and Z and risk of AMD [66, 67]. A systematic review and meta-analysis on this matter analyzed six longitudinal cohort studies and found that early and late AMD have different relationships with the intake of L and Z [68]. Dietary intake of these carotenoids was significantly related to a reduction in risk of late AMD (RR 0.74; 95 % CI 0.57, 0.97), and a statistically significant inverse association was observed between L/Z intake and neovascular AMD risk (RR 0.68; 95 % CI 0.51, 0.92). No significant relationship was found for dietary intake of these carotenoids and early AMD. In the Age-Related Eye Disease Study (AREDS) Report 22, the relationship between dietary intake of L/Z and late AMD was evaluated in 4519 patients. Dietary L/Z intake was inversely associated with neovascular AMD [odds ratio (OR) 0.65; 95 % CI 0.45–0.93], geographic atrophy (OR 0.45; 95 % CI 0.24–0.86), and large or extensive intermediate drusen (OR 0.73; 95 % CI 0.56–0.96), comparing the highest vs lowest quintiles of intake, after adjustment for total energy intake and nonnutrient-based covariates. Other nutrients (β-carotene, vitamin C, vitamin E, lycopene, etc.) were not independently related to AMD [16]. Furthermore, participants from the Rotterdam Study were enrolled into a case–control study investigating whether dietary nutrients could reduce the genetic risk of early AMD. Participants from a large population-based cohort at risk of AMD were followed for a mean of 8.6 years. They reported that high dietary intake of nutrients with antioxidant properties such as L and Z, β-carotene, omega-3 fatty acids, and zinc reduced the risk of early AMD in those at high genetic risk [69].

The first report that analyzed the association between MP density in the human retina and risk for AMD was published by Bone et al. [70]. It had demonstrated that retinas from donors with AMD showed significantly lower levels of MP compared to retinas without; and donors with the highest quartile of L/Z had an 82 % lower risk of having AMD compared to donors in the lowest quartile. This was the first study to report decreased levels of MP density in patients with AMD, which correlated with previous studies analyzing diet and serum carotenoid levels. The authors did note that decreased MP could at least in part be attributable to the disease destructive process. Lower levels of MP have also been associated with increased age and other risk factors for AMD, including a positive family history of AMD and smoking [71].

Several interventional studies have suggested that visual function of AMD patients can be improved by L and/or Z supplementation [72, 73], but some have still failed to find such results [74]. Liu et al. performed a meta-analysis comparing results of eight randomized, double-blind trials involving 1176 AMD patients in total, which compared L and/or Z intervention with placebo [75]. Four of seven studies demonstrated a significant improvement in visual acuity (VA) with supplementation. Moreover, patients supplemented with those xanthophyll carotenoids had significantly increased CS at all spatial frequencies compared with those who received placebo. The analysis demonstrated that oral supplementation is associated with significant improvements in VA and CS in a dose–response relationship. Also, a linear association of MPOD and an increase in VA and CS at middle frequency was noted, which suggested that morphological restoration of the macula might have been responsible for the observed effects of improved function. These results were in agreement with previous findings from the same group, who found significant morphological changes in MP at the central retina within 24 weeks, but no improvement in visual function until 48 weeks [76]. Similarly, the results of the TOZAL study also indicated that AMD patients were likely to require supplementations for at least 6-months to obtain positive changes in VA outcomes [77]. Patients with late AMD compared with early AMD tended to have a less significant improvement in VA, and this finding was attributed to the loss of macular photoreceptors in the late stage of the disease [75].

After the release of findings of smaller trials mentioned above, the Age-Related Eye Disease-2 Study (AREDS2) was published [17]. It was a multicenter, randomized, double-masked, placebo-controlled clinical trial following 4203 participants with intermediate AMD or large drusen in 1 eye and advanced AMD in the fellow eye for approximately 5 years. Participants were assigned to placebo, L (10 mg) and Z (2 mg), omega 3 fatty acids, or the combination of L, Z, and omega 3 fatty acids. In addition they were administered either the original AREDS formulation or some modification of the original formulation (eliminating β-carotene, lowering zinc dose, or a combination of the two). The original analysis did not find significant effects from the supplementation of these bioactive substances. However, a secondary analysis of the effects of L/Z on AMD progression in AREDS 2 revealed definitively positive results [78]. The authors re-evaluated the results of AREDS2 by analyzing L/Z vs no L/Z, and comparing L/Z vs β-carotene. In the analysis of L/Z vs no L/Z, the development of late AMD was significantly decreased in patients treated with L/Z. Analyses of the comparison of L/Z vs β-carotene also showed a significant decrease in the risk of developing late AMD and neovascular AMD in the L/Z group, but did not appear to influence the development of geographic atrophy. In analyses restricted to eyes with bilateral large drusen at baseline, the comparison of L/Z vs β-carotene showed protective effects of MP with regard to progression to late AMD and neovascular AMD. The authors concluded that the totality of evidence regarding beneficial and adverse effects of β-carotene in AREDS2 and other studies suggests that L/Z is more appropriate than β-carotene for the new AREDS2 formulation. It is important to emphasize that unlike other carotenoids, β-carotene has a much broader distribution within the body that correlates with the wide expression of the cleavage enzymes in various tissues. Also the mechanism of tissue uptake of β-carotene is not fully understood [26] and this may play a role on the absence of significant beneficial effects showed on AREDS 2.

Finally, recent trials showed positive results regarding retinal function after treatment with macular carotenoids. Huang et al. found significant improvements in retinal sensitivities of early AMD patients measured by multifocal electroretinography and microperimetry after 48 weeks of supplementation with L alone or L and Z [79]. Akuffo et al. compared the impact of sustained supplementation using different carotenoid formulations on MPOD and visual function (CS and VA) in early AMD. After 2 years of follow up, MP augmentation was superior in the groups receiving the 3 macular carotenoids and after 3 years, all groups had significant improvements in CS. No significant changes in VA or progression to advanced AMD were observed. These findings suggest that adding a higher proportion of MZ leads to a panprofile augmentation in MPOD values and improvements in CS, indicating that the inclusion of MZ may confer benefits for the treatment of early AMD [22, 80].

## Role of lutein and zeaxanthin in other retinal diseases

The encouraging results described above have led to subsequent investigations into the role of antioxidants in other diseases, including diabetic retinopathy (DR) and retinopathy of prematurity (ROP). Retinal ischemia can lead to neovascularization, hemorrhage and blindness. Oxidative stress plays a role in the pathogenesis of both conditions, and earlier evidence suggests that antioxidant supplementation may prevent disease progression [81].

ROP is a disease that can cause blindness in very low birthweight infants. The incidence of ROP is closely correlated with weight and gestational age at birth. Despite current therapies, ROP continues to be a highly debilitating disease. Oxygen has been well characterized with regard to its key role in retinal neoangiogenesis. Low or high levels of oxygen regulate the normal or abnormal production of hypoxia-inducible factor-1(HIF-1) and vascular endothelial growth factors, which are the predominant regulators of retinal angiogenesis [82]. The relatively avascular retina then becomes hypoxic with increasing metabolic demand, which initiates the expression of proangiogenic factors. This stimulates aberrant angiogenesis, leading to intravitreal neovascularization and its complications [82, 83]. Also, there is an imbalance between the generation and sequestration of reactive oxygen species, and the developing retina in premature infants is particularly susceptible to oxidative damage for several reasons. The high proportion of long chain poly-unsaturated fatty acids makes the retina susceptible to lipid peroxidation, which can damage the tissue. In addition, preterm infants have reduced levels of antioxidants compared to full-term infants, since they are often produced or accumulated later in gestation. Hence, in preterm infants, the endogenous antioxidant system is overwhelmed, leading to a pro-oxidative state, capable of causing irreversible damage to various cell structures [84]. Antioxidants can protect retinal cells from oxidative damage and have been shown to inhibit microvascular degeneration in animal models of ROP [85, 86]. A very recent study demonstrated that Z significantly inhibited the expression of VEGF and accumulation of HIF-1α protein caused by hypoxia in a primary culture of human retinal pigment epithelium cells [87]. Moreover, during fetal development, L is the dominant retinal carotenoid, and Z and MZ slowly accumulate with time [2]. Also, the presence of L in the umbilical cord at birth indicates that there is placental transfer with concentrations peaking in the third trimester [88]. The relative deficit of antioxidants in preterm infants and the growing evidence from animal studies suggest a possible role for antioxidant supplementation in the prevention of ROP progression.

A randomized controlled trial of 150 newborns demonstrated that neonatal supplementation of L in the first hours of life increased biological antioxidant potential and reduced levels of total hydroperoxide [89]. Four randomized controlled trials investigated the relationship between xanthophylls and ROP [90–93]. L was the primary xanthophyll used in the supplementation trials due to its predominance in the infant retina. Two multicenter placebo-controlled randomized clinical trials studying ROP prevention supplemented preterm infants with L/Z via oral feeds of maternal milk, donor human milk, or preterm formula [90, 91]. The studies could not find any significant differences in ROP incidence. In addition, while not statistically significant, supplemented subjects with ROP showed a 50 % decrease in progression from early to threshold ROP stages [91]. A third clinical trial investigated the effect of weight-base dosing, since AMD trials have suggested better outcomes with higher carotenoid doses. This trial did not show a difference in ROP incidence with weight-based doses, but the study was limited by small sample size [92]. The fourth multicenter randomized controlled trial compared plasma carotenoid levels in preterm infants fed formula with and without L, lycopene, and β-carotene to carotenoid levels in full-term infants fed human milk. The incidence of ROP was similar between the premature formula-fed groups, but the supplemented group had less progression to severe stages versus the control group. The supplemented group also had similar plasma L levels compared to full-term infants fed human milk. The study also compared L levels with photoreceptor activity and found that normal plasma L levels at 50 weeks of age correlated with saturated response amplitude in rod photoreceptors and rod photoreceptor sensitivity [93]. The authors concluded that carotenoid supplementation may decrease inflammation, and their results pointed to the protective effects of L on preterm retina health and maturation. To date no clinical trials have specifically tested the hypothesis that L affects ROP outcomes. While future supplementation trials monitoring long-term outcomes in ROP would be crucial, current evidence may suggest a role for carotenoid supplementation in the prevention of ROP and normal photoreceptor development in preterm infants.

In DR, prolonged hyperglycemia causes oxidative stress via several different pathways [15, 94]. Evidence from animal models suggests that L and Z can block the pathways leading to oxidative stress by quenching oxygen radicals, therefore preserving retinal function and preventing the development of DR [95, 96]. Animal studies have found that neuroprotective activities of L prevent neuronal loss in diabetic retinas [97, 98]. While a number of studies have examined the role of carotenoids in the development of diabetes mellitus (DM), there are a limited number of studies examining their role in the development of DR. A serum analysis of patients with type 2 DM demonstrated that patients with a higher concentration of L, Z, and lycopene compared to α-carotene, β-carotene and β-cryptoxanthin had a 66 % reduction in the risk of DR after adjusting for confounding variables [99]. A study published by Lima et al. demonstrated that diabetic patients had significantly lower levels of MPOD when compared to age-matched controls. In comparing the diabetic patients, those with retinopathy had even lower MPOD than subjects without, and those levels correlated with glycosylated hemoglobin [100]. Another study published by Hu et al. found that daily supplementation of non-proliferative DR patients with L/Z increased MPOD and improved VA, CS and macular edema when compared to placebo [101]. Evidence supporting the role of MP in the prevention and treatment of DR is currently limited, but animal models and some human supplementation trials suggest that there is a role for L and Z in reducing oxidative damage and possibly preventing disease progression.

## Conclusions

MP has been extensively studied during the past decades. Epidemiological studies have revealed that low MP levels are associated with higher risk of AMD. Several large observational studies demonstrated that high dietary intake and higher serum levels of L and Z are associated with a lower risk of AMD, especially late AMD. Randomized controlled clinical trials have revealed that supplementation of L and Z increases MPOD, improves visual function and decreases the risk of progression to late AMD, especially neovascular AMD. While the last two decades of research have provided many insights into the role of MP and other antioxidants in AMD, future research studies investigating the optimal antioxidant supplement dose, the role of early supplementation, the relationship between MPOD as a risk factor for disease onset and progression, and the impact of genetic risk factors are necessary to better understand the disease process and provide more therapeutic options to patients with AMD. Finally, current studies on the preventive and therapeutic effects of L and Z on ROP, DR and cataract have yielded varied results. Further investigations are necessary to fully understand the role of MP in the prevention and treatment of eye diseases such as AMD, ROP, DR, and cataract.

## Abbreviations

AMD: age-related macular degeneration
MP: macular pigment
MZ: meso-zeaxanthin
L: lutein
Z: zeaxanthin
ROS: reactive oxygen species
MPOD: macular pigment optical density
CS: contrast sensitivity
HFP: heterochromatic flicker photometry
VA: visual acuity
ROP: retinopathy of prematurity
DR: diabetic retinopathy
DM: diabetes mellitus
